# Draft Genome Sequences of Four Aeromonas salmonicida subsp. achromogenes Strains, 23051, 23053, 23055, and 23056, Isolated from Senegalese Sole (*Solea senegalensis*)

**DOI:** 10.1128/MRA.00631-19

**Published:** 2019-08-15

**Authors:** Antony T. Vincent, Alain Le Breton, Alex Bernatchez, Cynthia Gagné-Thivierge, Valérie E. Paquet, Eric Thibault, Steve J. Charette, Hubert Gantelet

**Affiliations:** a INRS-Institut Armand-Frappier, Bacterial Symbionts Evolution, Laval, Quebec, Canada; b SeLARL Vet’Eau, Grenade-sur-Garonne, France; c Institut de Biologie Intégrative et des Systèmes (IBIS), Université Laval, Quebec City, Quebec, Canada; d Département de Biologie, Faculté des Sciences et de Génie, Université Laval, Quebec City, Quebec, Canada; e Département de Biochimie, de Microbiologie et de Bio-informatique, Faculté des Sciences et de Génie, Université Laval, Quebec City, Quebec, Canada; f Ceva Biovac, Beaucouzé, France; University of Arizona

## Abstract

The bacterial species Aeromonas salmonicida officially has five subspecies. A large majority of the currently available sequences come from Aeromonas salmonicida subsp. salmonicida, which causes furunculosis in salmonids. We present the genomic sequences of four Aeromonas salmonicida subsp. achromogenes strains. This will help increase the robustness of genomic analyses for this subspecies.

## ANNOUNCEMENT

The bacterium Aeromonas salmonicida is divided into five officially recognized subspecies, A. salmonicida subsp. salmonicida, A. salmonicida subsp. smithia, A. salmonicida subsp. achromogenes, A. salmonicida subsp. masoucida, and A. salmonicida subsp. pectinolytica (1). Strains of all of these subspecies, with the exception of A. salmonicida subsp. pectinolytica, are aquatic animal pathogens and cause significant economic losses to the aquaculture industry around the world (2). Recent discoveries suggest a much greater diversity than was previously suspected for this species (3–5). Most of the genomes available come from strains of A. salmonicida subsp. salmonicida, making it difficult to perform robust comparative analyses and draw clear conclusions.

We present the draft genome sequences of four strains of *A. salmonicida* subsp. *achromogenes* (23051, 23053, 23055, and 23056), a subspecies that previously had only one publicly available genome, that of strain AS03, which was isolated from crucian carp (Carassius carassius) (6). The four strains were isolated from Senegalese sole (Solea senegalensis) in March 2014 (23056), January 2016 (23051), and May 2016 (23053 and 23055). From diseased fish, seeding on tryptic soy agar (TSA) was made from spleen, kidney, heart, and skin lesions using a sterile inoculation loop. The cultures were incubated for 48 to 72 h at 25°C. After the initial culture, a colony of the dominant population on the medium was transplanted to obtain a pure culture. The initial identification of these strains was carried out by matrix-assisted laser desorption ionization–time of flight (MALDI-TOF) mass spectrometry. These results indicated that their genus was *Aeromonas* but did not provide reliable data for the species.

For genomic DNA extraction, bacterial isolates were recovered from frozen stocks at −80°C, plated on TSA, and incubated at 18°C for 48 h. For each strain, several colonies were resuspended in 1 ml of tryptic soy broth, and genomic DNA was subsequently extracted from this bacterial suspension using DNeasy blood and tissue kits (Qiagen, Canada), according to the manufacturer’s instructions. The DNA was then used to make the libraries using a Kapa Hyper Prep kit and was sequenced by the Illumina MiSeq platform using 2 × 300-bp reads (IBIS, Université Laval). The resulting sequencing reads were verified with FastQC version 0.11.8 (http://www.bioinformatics.babraham.ac.uk/projects/fastqc/) and subsequently *de novo* assembled in contigs using SKESA version 2.3.0 (7). For all analyses, default parameters were used, unless otherwise specified. The statistics for the four assemblies are presented in Table 1. The sequences were annotated using the Prokaryotic Genome Annotation Pipeline (PGAP) (8) and deposited in GenBank.

**TABLE 1:** Sequencing and assembly metrics for the strains used in this study

Strain	Assembly size (bp)	No. of contigs	*N*_50_ value (bp)	GC content (%)	No. of CDS^*a*^	Coverage (×)	No. of reads	Assembly accession no.	SRA accession no.
23051	4,417,328	304	26,177	58.70	3,821	115	2,119,290	VCSC00000000	SRX6411896
23053	4,417,561	308	26,177	58.70	3,820	140	2,547,694	VCSB00000000	SRX6411897
23055	4,419,503	305	26,320	58.70	3,819	120	2,199,592	VCSA00000000	SRX6411898
23056	4,371,362	297	26,330	58.70	3,763	190	3,555,732	VCSD00000000	SRX6411895

a CDS, coding sequences.

Finally, taxonomic identification was performed by molecular phylogeny coupled with an average nucleotide identity (ANI) analysis, using a previously published method and data set (5). A matrix of percentage of conserved proteins (POCP) values (9) was also calculated with GET_HOMOLOGUES version 20190102 (10) to determine more thoroughly the identities of the strains.

**FIG 1:**
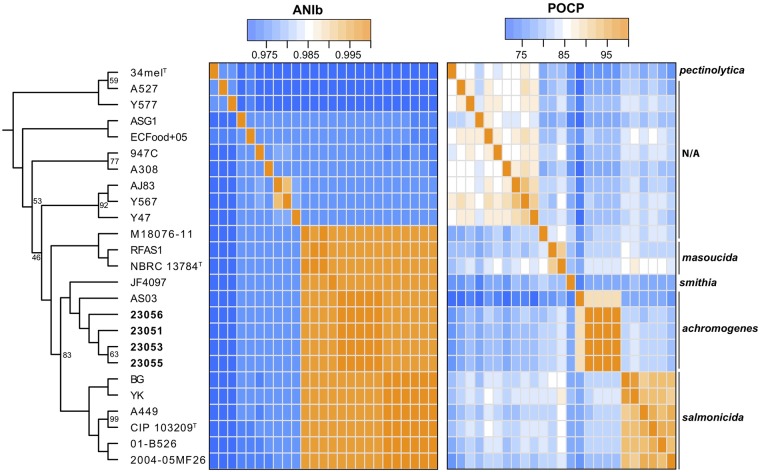
A dendrogram coupled to a matrix with ANI values and a matrix with POCP values (calculated using the OMCL algorithm through GET_HOMOLOGUES [10]). The phylogenetic tree was made from sequences of 1,952 orthologous genes (determined with GET_HOMOLOGUES) corresponding to 1,764,800 positions. Only bootstrap values less than 100 are shown. The four strains described in this study are in bold. The data set and bioinformatics procedures are published elsewhere (5). N/A, not applicable.

Molecular phylogeny has shown that the four strains whose genomes have been sequenced are found in the clade formed by A. salmonicida subsp. smithia and A. salmonicida subsp. achromogenes (Fig. 1). However, as described previously in the literature (3–5), the ANI values do not make it possible to discriminate between A. salmonicida subsp. masoucida, A. salmonicida subsp. smithia, A. salmonicida subsp. achromogenes, and A. salmonicida subsp. salmonicida. However, the POCP values made it possible to trace the boundaries between the different subspecies and to show that the four strains are of A. salmonicida subsp. achromogenes.

### Data availability.

The genome sequences of the four *A. salmonicida* subsp. *achromogenes* strains have been deposited in DDBJ/ENA/GenBank under the following accession and BioSample numbers: VCSC00000000 and SAMN11836205 for 23051, VCSB00000000 and SAMN11836206 for 23053, VCSA00000000 and SAMN11836207 for 23055, and VCSD00000000 and SAMN11836204 for 23056, respectively.
